# Effect of Duration of Exposure to Cement Dust on Respiratory Function of Non-Smoking Cement Mill Workers

**DOI:** 10.3390/ijerph10010390

**Published:** 2013-01-16

**Authors:** Sultan Ayoub Meo, Abdul Majeed Al-Drees, Abeer A. Al Masri, Fawzia Al Rouq, Muhammad Abdul Azeem

**Affiliations:** 1 Department of Physiology, College of Medicine, King Saud University, P.O. Box 2925, Riyadh, 11461, Kingdom of Saudi Arabia; 2 Department of Physiology, Faculty of Health and Medical Sciences, Hamdard University, Karachi-74600, Pakistan; 3 Department of Physiology, Dewan Medical College, Karachi-74900, Pakistan

**Keywords:** air pollution, cement dust, lung function, occupational hazards

## Abstract

This study aimed to determine the effect of long term exposure to cement dust on lung function in non-smoking cement mill workers. This is a cross-sectional study of respiratory functions. Spirometry was performed in 100 apparently healthy volunteers; 50 non-smoking cement mill workers and 50 non-smoking un-exposed subjects. Based on the duration of exposure, cement mill workers were divided into three groups, less than 5, 5–10 and greater than 10 years. All subjects were individually matched for age, height, weight, and socioeconomic status. Pulmonary function test was performed by using an electronic spirometer. Significant reduction was observed in the mean values of Forced Vital Capacity (FVC), Forced Expiratory Volume in one second (FEV_1_), Peak Expiratory Flow (PEF) and Maximal Voluntary Ventilation in cement mill workers who had been working in the cement industry for more than 10 years compared to their matched un-exposed group. Lung functions in cement mill workers were significantly impaired and results show a long term duration response effect of years of exposure to cement dust on lung functions.

## 1. Introduction

The worldwide community, especially the people in developing countries, is facing increasing risks of respiratory diseases due to production of smoke and dust in different occupational and industrial sectors [1,2]. The health risks posed by inhaled dust particles are influenced by the duration of exposure and the biological responses exerted by the particles [3]. Cement industry is one of the largest manufacturing industries and its workers are exposed to dust at various manufacturing and production processes [4]. Portland cement dust is a mixture of calcium oxide, silicon oxide, aluminum tri oxide, ferric oxide, magnesium oxide, sand and other impurities [5]. The aerodynamic diameter of cement dust particles is within the respirable extent [6], consequently occupational exposure to cement dust can cause numerous health hazards including the onset of acute or chronic respiratory diseases and respiratory function deficits. 

Lung function impairment is the most common occupational respiratory problem in subjects exposed to dust in industrial sectors [2]. Studies are available on lung function and cement dust, but most of these studies were conducted without considering the long term duration-response effect between years of exposure and respiratory function impairment [7] and were not explained by promising physiological factors which greatly influence the lung function such as age, height, weight, ethnicity, and socioeconomic status. The present study has attempted to minimize mystifying interpretational factors by using matched controls, excluding smokers and workers with previous industrial exposure other than cement industries. Moreover, occupational and respiratory physicians should know the magnitude of problem of lung function impairment with duration of exposure to cement dust. Therefore, this study aimed to determine the effects of length of exposure to cement dust on lung function and also provide information to cement mill workers about the hazards of cement dust on lung function and measures for its prevention. 

## 2. Subjects and Methods

### 2.1. Subjects

This cross sectional study was conducted under the supervision of Department of Physiology, Faculty of Health and Medical Sciences, Hamdard University, Karachi, Pakistan. In this study, 105 cement mill workers, working in a same cement company, were selected. A detailed interview was conducted followed by history and clinical examination to determine whether they would be included in the study or not. All the participants were questioned with regards to smoking cigarettes and other tobacco products. After the initial interviews, clinical history and examination, 50 apparently healthy, volunteer, male cement mill workers with age 36.86 ± 1.50 years (mean ± SEM; range 20–60) years were selected. The participants were further divided into three groups based on the duration of exposure less than 5, 5–10 and greater than 10 years. The participants worked for at least 8–10 hr a day for six days per week, without using adequate respiratory protective measures such as mouth pieces, goggles. These 50 cement mill workers were matched with another group called un-exposed (control) group. The control group was selected in a similar way. Initially, 80 subjects were interviewed, clinical history and examination were conducted, finally 50 matched volunteer, healthy men, mean age 37.80 ± 1.66 years (mean ± SEM, range 20–60 years) were selected. The control group was primarily composed of clerical staff, shopkeepers and salesmen. It was kept in mind that the shops of the shopkeeper must not be in congested areas, or motor vehicle polluted areas. All subjects were individually matched for age, height, weight, and socioeconomic status. The Institutional Review Committee, Board of Advanced Studies and Research, Faculty of Health and Medical Sciences, Hamdard University, Karachi, Pakistan approved the study (COMD-097). 

### 2.2. Exclusion Criteria

Subjects with deformities of the thoracic cage, vertebral column, musculoskeletal system, known cases of neuromuscular diseases, gross anemia, diabetes mellitus, chronic obstructive pulmonary diseases, malignancy, drug addicts and cigarette smokers were excluded. The subjects who were taking vigorous exercise regularly or had undergone abdominal, or chest surgery, and were working in any industry other than cement industry were excluded from the study. 

### 2.3. Methods: Spirometry

Spirometry was performed by using an electronic spirometer (Compact Vitalograph, UK). All respiratory function tests were carried out at a fixed time of the day to minimize the diurnal variation [8]. The apparatus was calibrated daily and operated within the ambient temperature range of 20–25 °C. The defined techniques in executing various lung function tests for the present study were based on the operation manual of the instrument with special reference to the official statement of the American Thoracic Society of Standardization of Spirometry [9]. After taking a detailed history and anthropometric data, the subjects were informed about the whole maneuver. The subjects were encouraged to practice this maneuver before doing the pulmonary function test. The test was performed with the subject in the standing position without using a nose clip. The test was repeated three times after adequate rest of 5 min to avoid the exertion. The results were obtained available in the spirometer. These parameters included Forced Vital Capacity (FVC), Forced Expiratory Volume in First Second (FEV_1_), Forced Expiratory Ratio (FEV_1_/FVC), Forced Expiratory Flow (FEF_25__–75%_) and Maximum Voluntary Ventilation (MVV). 

### 2.4. Statistical Analysis

The data were entered into the computer and analyzed by using the Statistical Package for Social Sciences (SPSS) version 10.0 programs for Windows. Unpaired Student’s *t*-test was applied to test the difference of the means between the two quantitative variables. The level of significance was achieved at p < 0.05. 

## 3. Results

The results are presented according to duration of exposure to cement industry (less than 5, 5–10, and more than 10 years). The statistical comparisons of the matching variables (age, height, and weight) were similar for both groups; hence the statistical validation of this information is not discussed. 

### 3.1. Duration of Exposure Less Than Five Years

Table 1 summarizes the comparison of the lung function parameters between cement mill workers and their matched control group. There was a significant decline for FVC in cement mill workers. However, no significant difference between the means of any other lung function data was observed between the groups. The mean duration of exposure in cement mill workers was 3.3 ± 0.26 years (mean ± SEM), range 2–4 years.

**Table 1:** Anthropometric and lung function data for cement mill workers with duration of exposure less than five years compared with their matched controls.

Parameters	Cement mill workers (mean ± SEM) (n = 10)	Control Subjects (mean ± SEM) (n = 10)	*p*-value
Age (years)	25.20 ± 2.33	26.50 ± 2.88	N S
Height (cm)	166.90 ± 1.61	168.10 ± 1.37	N S
Weight (kg)	60.70 ± 2.74	62.70 ± 3.18	N S
FVC (litres)	3.40 ± 0.33	4.25 ± 0.33	0.05
FEV_1_ (litres)	2.86 ± 0.29	3.35 ± 0.26	NS
FEV_1_/FVC (%)	84.8 ± 3.29	79.1 ± 3.74	NS
PEF (litres/min)	313.4 ± 35.24	357.7 ± 41.00	N S
FEF_25__–75%_ (litres/s)	3.62 ± 0.48	3.92 ± 0.43	N S
MVV (litres/min)	107.3 ± 11.04	125.9 ± 10.03	N S

Values are presented as Mean ± SEM; N S = non-significant.

### 3.2. Duration of Exposure 5–10 Years

Cement mill workers exposed for 5–10 years, showed a significant reduction in FVC, FEV_1 _and MVV compared with their matched controls (Table 2). However, these workers did not show a significant reduction in FEV_1_/FVC, PEF and FEF_25__–75%_ relative to controls. The mean duration of exposure in these cement mill workers was 7.30 ± 0.47 years (mean ± SEM), range 6–10 years. 

**Table 2:** Anthropometric and lung function data for cement mill workers with duration of exposure 5–10 years compared with their matched controls.

Parameters	Cement mill Workers (mean ± SEM) (n = 10)	Control Subjects (mean ± SEM) (n = 10)	*p*-value
Age (years)	32.70 ± 3.40	34.30 ± 3.75	N S
Height (cm)	166.20 ± 2.00	165.30 ± 2.26	N S
Weight (kg)	62.90 ± 2.51	66.10 ± 2.18	N S
FVC (litres)	3.11 ± 0.22	4.28 ± 0.11	0.0005
FEV_1_ (litres)	2.61 ± 0.22	3.24 ± 0.12	0.01
FEV_1_/FVC (%)	85.50 ± 5.96	76.00 ± 2.88	N S
PEF (litres/min)	318.7 ± 44.11	353.8 ± 32.42	N S
FEF_25__–75%_ (litres/s)	3.72 ± 0.35	3.51 ± 0.32	N S
MVV (litres/min)	98.10 ± 8.57	121.60 ± 4.56	0.01

Values are presented as Mean ± SEM; NS = non-significant.

### 3.3. Duration of Exposure More Than 10 Years

Cement mill workers exposed for more than 10 years showed a significant reduction in FVC, FEV_1_, MVV, and PEF relative to their matched controls (Table 3). However, there were no significant differences in FEV_1_/FVC and FEF_25__–75%_ data relative to controls. The mean duration of exposure in these cement mill workers was 18.0 ± 0.70 years (mean **±** SEM), range 11–28 years. 

**Table 3:** Anthropometric and lung function data for cement mill workers with duration of exposure more than 10 years compared with their matched controls.

Parameters	Cement mill workers (mean ± SEM) (n = 30)	Control Subjects (mean ± SEM) (n = 30)	*p*-value
Age (years)	42.13 ± 1.37	42.73 ± 1.75	N S
Height (cm)	165.03 ± 1.17	164.27 ± 1.19	N S
Weight (kg)	64.97 ± 1.97	63.27 ± 1.34	N S
FVC (litres)	3.13 ± 0.14	3.87 ± 0.10	0.0005
FEV_1_ (litres)	2.41 ± 0.11	2.87 ± 0.08	0.0005
FEV_1_/FVC (%)	79.00 ± 3.01	74.70 ± 1.72	N S
PEF (litres/min)	283.57 ± 24.78	365.70 ± 20.67	0.01
FEF_25__–75%_ (litres/s)	2.98 ± 0.22	3.04 ± 0.18	N S
MVV (litres/min)	90.66 ± 4.23	107.83 ± 3.17	0.005

Values are presented as Mean ± SEM; NS = non-significant.

## 4. Discussion and Conclusions

### 4.1. Main Findings

Occupational and environmental exposure to cement dust and their effects on human health is a leading respiratory health problem. Exposure to cement dust can cause various acute and chronic respiratory diseases including respiratory function impairment. Literature is available on respiratory function and cement dust, but the majority of the studies were conducted without considering the association with a long term duration-response effect between years of exposure and pulmonary function and were not elucidated by promising physiological factors which greatly influence the lung function such as age, height, weight, ethnicity, smoking and socioeconomic status. 

The present study found a duration response effect and shows that long term exposure to cement dust prominently decreased the pulmonary function. Cement mill workers with duration of exposure greater than 10 years showed a significant reduction in FVC, FEV_1_, PEF and MVV relative to their matched controls. In contrast, Shamssain and Thompson [10] reported that the mean values for FVC and FEV_1_ were not significantly decreased in cement mill workers. They also did not show a significant relationship between the length of exposure in cement industry and lung function parameters. A possible reason for this difference could be that, Shampassian and Thompson [10] did not appropriately match the anthropometric parameters especially height, which were significantly different (p < 0.025) between groups. In addition, they did not report about calibration of the apparatus, which is an essential and important factor to obtain the precise results.

Al-Neaimi *et al.* [11] demonstrated that the ventilatory function (FVC, FEV_1_, and PEF) were significantly lower in the cement mill workers compared with unexposed subjects. Meo *et al.* [12] conducted a study on lung function and Surface Electromyography of intercostal muscles in cement mill workers. The study population were closely matched in terms of anthropometric variables and found significant reduction in lung function parameters, FVC, FEV_1_, PEF and MVV in cement mill workers compared with controls, however, they did not report association between lung function and duration of exposure to cement dust. Similarly, Mwaiselage *et al.* [13] investigated ventilatory function in cement factory workers and reported that exposed workers had significantly lower FVC, FEV1, and PEF than controls. Nordby *et al.* [14] reported that Forced Expiratory Volume in the first second (FEV_1_) reduced with an exposure-response relationship in the highest compared with the lowest exposure level of cement dust. Concurrently, Zelke *et al.* [15] found that FVC, FEV_1_ were significantly reduced among the cement production workers but not among the controls. The reduction in lung function was probably associated with high cement dust exposure. 

In parallel to our findings, Merenu *et al.* [16] investigated the effect of cement dust exposure on 56 cement factory workers with a mean of 10 years exposure to cement dust on lung function. They found that the vital capacity and forced expiratory volume in one second were significantly lower in cement factory workers than in control subjects. Their results suggest that chronic cement dust exposure impairs lung function. Similarly, El Badari and Saeed [17] reported a significant reduction in FVC, FEV_1_ and PEFR in cement dust exposed workers compared to control. The lung function indices were found to be reduced with increasing duration of exposure to cement dust. Our results are in conformity with these results.

Olerue [5] reported that the lung function parameters FVC and FEV_1_ were decreased with duration of employment in cement industry, but this level was not statistically significant. Our results, however shows a significant decrease in FVC and FEV_1_ with increased duration of occupational exposure to cement dust (Table 1, Table 2 and Table 3). A possible reason for this difference is the selection criteria. Olerue [5] selected 76 cement mill workers from a cement plant that had started production of cement only 6 years ago and grouped the cement mill workers with very little duration of exposure, into 2 groups, namely 6–36 months and 37–72 months. This duration of occupational exposure was small and it could be the reason for the non-significant difference in the lung function parameters FVC and FEV_1_. 

At the differences, Fell *et al.* [7] found that the mean pulmonary function indices were similar for cement mill workers and control group. There was no duration-response-related decrease in lung function indices. However, in the present study we found a decline in lung function parameters with period of exposure to cement dust. The main reason for this difference is the selection protocol of the cement mill workers and the control group. Fell *et al.* [7] selected 119 cement mill workers, from them only 19 were non smokers; remaining all were either smokers or ex-smokers. They selected the control group from an ammonia producing industry. They compared the lung function parameters of cement mill workers with a control group of subjects employed in ammonia industry. It is an established fact that FVC and FEV1 decrease significantly among cigarette smokers [18] and exposure to ammonia [19]. However, in the present study we entirely excluded the smokers and our control group was composed of non-smoking clerical staff, shopkeepers and salesmen, all subjects were individually matched for age, height, weight, and socioeconomic status. Based on these reasons Fell *et al.* [7] results are in contrast to our findings.

Alakija * et al.* [20] showed that cement mill workers had a consistent decline in FVC, FEV_1_ and PEF with prolonged years of service in the cement industry. They also reported that workers who had less than five years of occupational exposure to the cement dust had a significantly higher FVC, FEV_1_ and PEF than the workers who had more than 15 years of exposure. The results of the present study are in agreement with the outcomes of Alakija *et al.* [20].

### 4.2. What This Study Adds

The present study adds evidence that cement dust adversely affects the respiratory function and this impairment is associated with duration of exposure to cement dust. The findings are of importance in that it highlights the needs to overcome the effects of long term exposure. This study also exhibits the magnitude of the effect in a population. It is advisable that health risk should be reduced by the collaboration between health officials, cement mill workers and the management of cement industry to adopt technical preventive measures, such as ventilated work areas and utilization of appropriate respiratory protective measures including dust preventive nose and mouth filters/masks and safety goggles. It is also suggested that workers must undergo pre-employment and periodic medical examinations including lung function test. We cannot deny the role of cement industry in the infrastructure, but we also believe that human health is more important and it cannot be compromised. Therefore, long term exposure to cement industries should be evaded and workers must be given official days off at least two times in a week and two months in a year. It is also recommended that the industry management should arrange health safety and education sessions biannually for the workers to provide awareness.

### 4.3. Study Limitations

The limitations of the present study include lack of specific dust exposure assessment and the relatively small sample size.
